# Development and validation of a health literacy scale for family caregivers of older people with chronic illness

**DOI:** 10.1186/s12912-024-02057-x

**Published:** 2024-07-01

**Authors:** Patrick Pui Kin Kor, Clare Tsz Kiu Yu, Yaqin Li, Alex Pak Lik Tsang, Lexi Han Zhi Tan, Simon Ching Lam, Paul Hong Lee, Justina Yat Wa Liu, Angela Yee Man Leung, Ka Ching Lee

**Affiliations:** 1 School of Nursing, The Hong Kong Polytechnic University, Hong Kong, China; 2 Division of Psychiatry, University of College London, London, UK; 3 School of Nursing, Tung Wah College, Hong Kong, China; 4 Southampton Clinical Trials Unit, University of Southampton, Southampton, UK; 5 United Christian Hospital, Hong Kong, China

**Keywords:** Health literacy, Caregiver, Older people, Psychometric validation

## Abstract

**Background:**

Family caregivers (FCs) encounter a variety of health problems in older people with chronic illness, necessitating a certain level of health literacy to access, understand, appraise and apply health information and services. This study aimed to develop and validate a scale for measuring health literacy among FCs of older people with chronic illness.

**Methods:**

Concept mapping was first employed to develop a conceptual model of health literacy of FCs. Scale domains were derived from the conceptual model, and item generation was performed using deductive and inductive methods. Quantitative methods, including merging scale dimensions and items, expert reviews, cognitive interviews, and item reduction analysis, were used to refine the scale. Confirmatory factor analysis was employed to validate the scale’s structure. Concurrent validity, internal consistency, and test-retest reliability were also examined.

**Results:**

A 20-dimension conceptual model was developed, and 60 items were generated for the scale. Expert review (content validity index > 0.85) and cognitive interview with FCs confirmed the relevance and clarity of the majority of the generated scale items. Confirmatory factor analysis with 451 FCs of older people with chronic illness supported a 5-factor structure (symptom management, daily personal care and household tasks, care coordination, communication and relationship with the care recipient, and self-care of caregivers) with 42 finalized scale items, including four levels of health literacy skills (accessing, understanding, appraising and applying health information). Concurrent validity with the European Health Literacy Questionnaire (HLS-EU-Q47) was satisfactory (*r* = 0.67, *p* < 0.01). The Cronbach’s *α* coefficient of the scale was 0.96, with subscales ranging from 0.84 to 0.91. The two-week test-retest reliability was 0.77 (*p* < 0.01).

**Conclusion:**

This study developed a conceptual model explaining the concept and factors of health literacy among FCs of older people with chronic illness that could provide the groundwork for future studies in developing relevant evidence-based interventions. A new Health Literacy Scale-Family Caregiver (HLS-FC) with satisfactory psychometric properties was developed in this study, which can be utilized to identify caregivers with insufficient health literacy and facilitate timely interventions by healthcare professionals.

**Supplementary Information:**

The online version contains supplementary material available at 10.1186/s12912-024-02057-x.

## Background

The global trend of population aging continues to increase each year. With advancements in medical care, the longevity of the older population is also increasing, leading to a higher incidence of chronic diseases and comorbidities [1]. Over 94.9% of older adults aged 60 or above have suffered from at least one chronic disease, with 78.7% coping two or more, where much of the care responsibility falls to their family members [2, 3]. Family caregivers (FCs) are often required to handle a wide range of health problems of older people, including supporting their activities of daily living (ADL) and/or instrumental activities of daily living (IADL), communicating with medical professionals, making health-related decisions, maintaining relationship with the care recipient, managing behavioral and psychological problems of the care-recipient [4–6]. Consequently, health literacy (HL) became an essential skill among FCs [7].

HL is defined as the knowledge, motivation, and competence to access, understand, appraise, and apply health information to make informed judgments and decisions in everyday life related to health care, disease prevention, and health promotion [8]. HL is also considered as a multidimensional construct including interpersonal factors, individual competencies, community, and health system factors [9]. Low HL among FCs can negatively impact on the care delivery and the health outcomes of care recipients [9]. Difficulties in comprehending health information and ineffective communication with health professionals are prevalent in people with inadequate HL, leading to a higher incidence of undetected health problems [10, 11]. This situation can be exacerbated by the negative aging stereotypes held by many young healthcare professionals [12]. In addition, unclear and insufficient health information is a persistent challenge for FCs in providing care, affecting the wellbeing of both patients and caregivers across physical, psychosocial, and spiritual domains. Previous studies have showed that inadequate HL is associated with increased utilizations of emergency medical service, higher rates of hospitalization, poorer quality of life, and delayed disease detection [8, 13, 14]. Therefore, HL is considered a modifiable risk factor for health disparities and an essential skill for maintaining the health of individuals and the community [15].

The critical barriers to identifying FCs with low HL are the lack of a conceptual model explaining the concept of HL in FCs and a validated measurement tool. Although conceptual models of HL have been developed, such as the Distributed Health Literacy Model [16] and the Health Literacy Pathway Model [17], they tend to focus primarily on patients. Thus far, only one conceptual model has been identified in the context of cancer caregiving, emphasizing the importance of accessing and comprehending health-related information, the relationship between caregivers, cancer patients, and healthcare providers, the utilization of support systems, and the management of caregiving challenges [18]. While the Conceptual Model of Cancer Caregiver Health Literacy offers unique insights into the concept of HL within the context of caregiving, there are conceptual distinctions between caregiving for cancer patients and older people with chronic illnesses. Older people, especially those aged 65 and above, are at an increasing risk of developing multimorbidity [19], where FCs of older people may face unique challenges and complexities in accessing and comprehending information related to symptoms management [20]. Additionally, FCs of older people often need to take on critical roles in decision-making regarding the utilization of health services due to the challenges faced by older adults, such as dementia, which leads to progressive disability and dependency [21]. Furthermore, FCs encounter distinct caregiving challenges. They often assume multiple care roles simultaneously, including caring for children or adolescents, parents, or multiple generations, which significantly increases their care burden. For example, family dynamics have been found to be an influential factor in the care burden experienced by FCs [22].

However, the literature review reveals a lack of HL measurement tools specifically designed for FCs of older people with chronic illness. There are two common HL measures: the Health Literacy Questionnaire (HLS) and the European Health Literacy Questionnaire (HLS-EU-Q) [23, 24]. While both measures are widely cited and have excellent psychometric properties [25, 26], they were not specifically developed for FCs of older people. Some HL tools have been developed for specific populations, such as the Health Literacy of Caregivers Scale-Cancer (HLCS-C) for cancer caregivers. There are also self-reported HL tools focusing on an older population; however, they were not tailored for caregivers [27, 28]. These HL measures, while valuable, do not capture the unique needs of HL in the context of FCs of older people. FCs of older people often face challenges related to multimorbidity and age-related diseases that require complex healthcare and community service support [19, 20]. Further, FCs often assume the primary responsibility to make health-related decisions for the care recipient, partly due to the older care recipient often suffering from conditions that adversely impact cognitive functioning, such as dementia [21]. Consequently, low levels of HL in this population have been linked to poor outcomes for both FCs and care recipients, emphasizing the importance of HL in this unique population [9]. Therefore, developing an HL tool specifically for this population would allow for accurately assessing the HL levels of these caregivers and facilitate evidence-based interventions that target HL for this population.

## Aim

In this study, we (1) developed a conceptual model; and (2) constructed and validated a new scale to measure HL for this population.

## Methods

This study consisted of two parts. The first part was the development of a conceptual model of HL among FCs of older people with chronic illness. The second part was the development and validation of an HL scale for this population. A validity-driven approach was employed to develop the scale based on the conceptualized model in part one.

### Part one: Development of the conceptual model

#### Concept mapping

Concept mapping is a participatory mixed-methods approach for identifying and organizing ideas on a topic of interest [29, 30]. The steps of concept mapping include selecting participants, generating statements, structuring statements, analyzing data, and interpreting data [31]. Trochim [31] recommended that a sample between 10 and 20 would typically provide sufficient information to perform concept mapping. To generate the statements, focus group interviews (5 groups, *n* = 30) were conducted six participants per group to reach data saturation [32].

#### Participants and settings

FCs were recruited through caregiver support groups at District Elderly Community Centers in Kowloon district, Hong Kong, online social media groups for caregivers (e.g., Facebook), and mass media outlets (e.g., newspapers and public health promotion talks). The eligibility criteria were (1) individuals aged 18 or above, (2) individuals who are currently taking care of family members (related by blood or marriage, such as spouses, parents, and grandparents) aged 60 or above with one or more chronic diseases, which require assistance to perform ADL and/or IADL, and (3) individuals who have been providing care to the care-recipient for at least four hours per week in the past six months (to ensure the caregiver are engaged with caregiving tasks) [33, 34].

#### Procedures

Purposive sampling was adopted to recruit caregivers with diverse socio-demographic backgrounds and caregiving patterns [35]. The participants’ eligibility was first screened by a trained research assistant under the supervision of a researcher. After fulfilling the inclusion criteria, participant selection was made by stratifying based on genders, age groups, and employment statuses, as employment status can significantly impact caregiving patterns [36]. After, selected participants were informed about the research purpose, and written consent were obtained. The focus group interviews took approximately 90 to 120 min. The first focus group aimed to generate statements from FCs about their HL in caring for older people, while the second focus group aimed to collect their comments about structuring the statements. A cash coupon was given to each caregiver to compensate them for their time and travel expenses.

#### Generation of statements and structuring of statements

In the focus groups, we asked FCs broad questions covering their experiences of taking care of their health and that of the care recipients, and how they obtain and use health information to make decisions. Semi-structured questions were asked to guide the discussion. Based on the items generated from the focus group interviews, participants were asked to sort the statements into clusters in a way that made sense to them [31]. They were asked to rank and rate the items using a 5-point Likert scale: (1) “relatively unimportant”, (2) “somewhat important”, (3) “moderately important”, (4) “very important”, and (5) “extremely important”.

#### Data analysis and interpretation

**Fig. 1:**
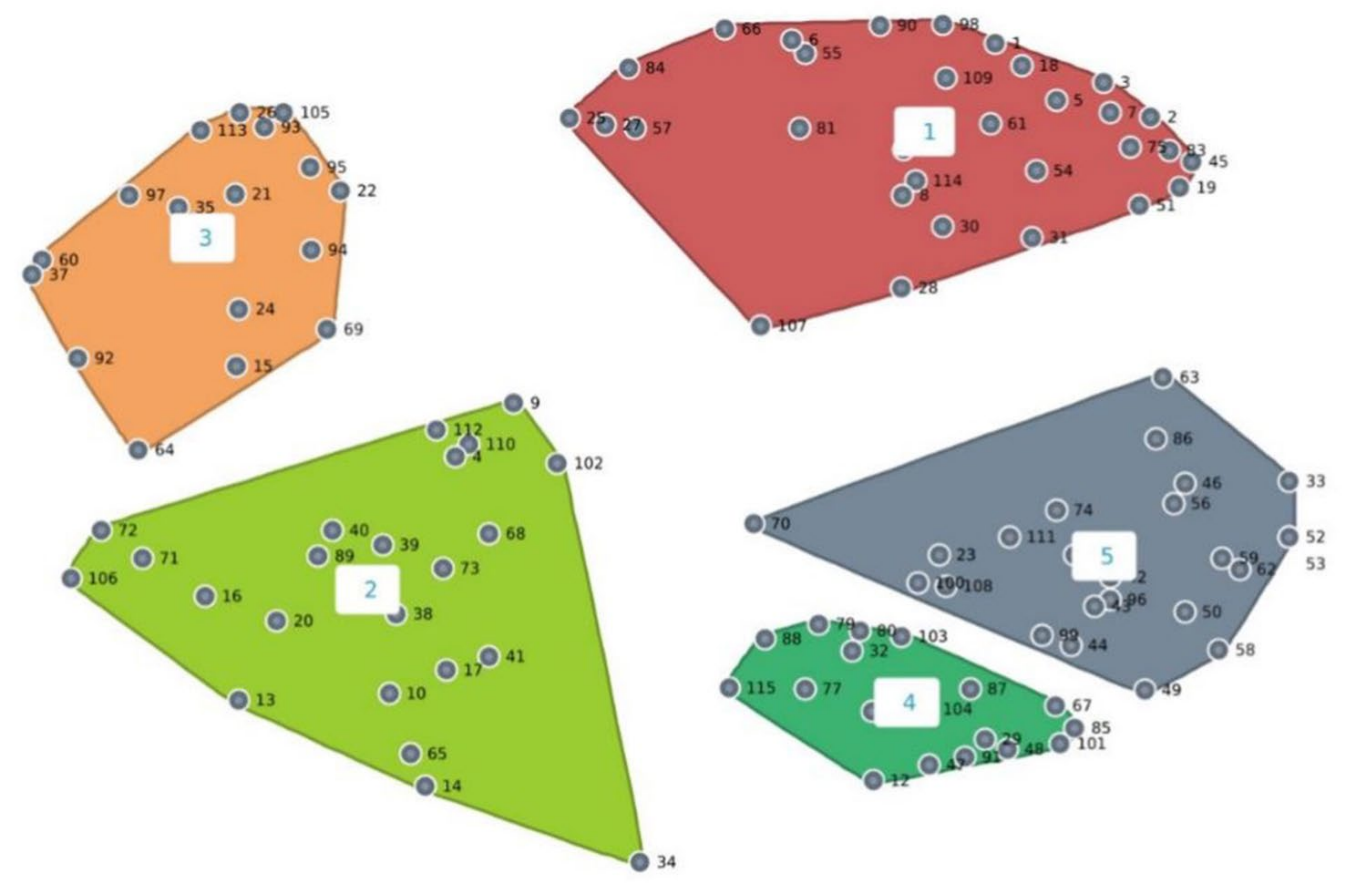
Two-dimensional representation of the combined statement groupings

All focus group interviews were digitally audio-recorded with participants’ consent, and field notes were taken to capture non-verbal information or cues. The information obtained from the focus groups, including the statements and the sorted data, was entered into the Concept System software (Groupwisdom™). A two-dimensional multidimensional scaling (MDS) analysis and hierarchical cluster analysis (using Ward’s algorithm) was performed to generate a concept map presenting a visual form of a two-dimensional representation of the combined statement groupings (Fig. 1). After generating the concept map, two researchers (Kor, Yu) independently examined the clusters represented in the concept map to (1) identify inappropriate statements on the map and re-assign each such statement into a different cluster that better represents its conceptual meaning; and (2) identify clusters with multiple concepts that may need to be split up [31]. To ensure trustworthiness, the researchers discussed the findings and label each cluster to create the concept map until consensus was reached.

### Part two: Development and validation of the HL scale

#### Identification of domains

A validity-driven approach was employed in developing the HL scale. A domain refers to the concept, attribute, or unobserved behavior that we aimed to identify [37]. Since the conceptual framework of holistic HL in FCs of older people with chronic illness was developed in part one, the following considerations were used to determine whether the domains should be included in the scale: (1) the domain should capture the experiences of caregivers caring for the recipients with a wide range of characteristics; (2) the domain should capture caregiving experiences in different levels and forms of support; and (3) the domain should align with the definition of caregivers in accessing, understanding, appraising, and using health information to promote and maintain the health of the care-recipient [18].

#### Item generation

After identifying the domains, a pool of items was generated through deductive and inductive methods. The deductive method was based on a description of the relevant domain. Thus, the statements on HL from the focus groups and conceptual model (developed in part one) were used to generate the items. Two researchers (Kor, Yu) independently reviewed the 219 entries generated from the focus groups, removed redundant statements, and grouped them into the smallest units. In addition, we consulted existing instruments of HL, such as the European Health Literacy Questionnaire (HLS-EU-Q47), as well as existing models of health literacy (e.g., the Integrated Conceptual Model of Health Literacy) during the item generation process [8, 24]. To ensure that the scale can differentiate low, moderate, and high levels of HL among FCs, the four cognitive skills of accessing, understanding, appraising, and applying health information were adopted to guide the selection of a set of items for each content area.

#### Expert review

Utilizing the dimensions and items developed in the above steps, an expert review was conducted after the quantitative merging of scale dimensions and items to establish content validity. Content validity, which is the degree to which an instrument has an appropriate sample of items for the construct being measured, is an essential criterion in scale development. It has been suggested that five to seven experts are sufficient to establish content validity through an expert review [37]. Our team of experts included one geriatrician, one general practitioner, two geriatric nurses, two social workers specializing in caregiving, and one health researcher specializing in HL. Three-point Likert scales (“low, moderate, high” and “unclear, neutral, clear,” respectively) were used to evaluate content validity. Items with a content validity index (CVI) score of less than 0.78 would be considered for revision or deletion [38]. An open-ended question was used to ask the experts if they have any further suggestions on the items.

#### Cognitive interview

Cognitive interviews were conducted to determine (1) whether FCs could interpret the questions as intended, (2) whether the items were relevant to the caregivers’ context, and (3) whether the caregivers encountered difficulties in responding to the items. A convenience sample of 12 participants was recruited from a caregiver support group from a District Elderly Community Center with the same eligibility criteria in part one above. Three rounds of cognitive interview (3 to 5 caregivers in each round) were carried out. The cognitive interviews were carried out to achieve full agreement [37]. The comments and suggestions collected from the focus group would be discussed by the research team for further revision of the items in the scale.

#### Scale validation

After refining the items from the cognitive interview, the proposed Health Literacy Scale-Family Caregiver (HLS-FC) was sent out for further validation. JC Nunnally [39] recommended that a minimum of 10 participants per scale item is required to perform factor analysis. The inclusion criteria were identical to those described in part one. Invitations were sent out to FCs via caregiver support groups at District Elderly Community Centers, online social media groups for caregivers (e.g., Facebook), and mass media outlets (e.g., newspapers and public health promotion talks).

#### Item reduction analysis

Item Response Theory (IRT) was adopted for item reduction analysis to ensure that only parsimonious and functional items would be retained in the scale [40]. The reduction was based on missing rate and mean in each item.

First, items with a missing rate higher than 5% were excluded. Missing rate was utilized as one of the criteria for the item reduction process, as unclear or ambiguous items tend to have a higher chance of non-response issues [41]. Mean values of each item were also taken into account during the item reduction process because extreme mean values may not provide the information as intended [42]. Considering the lack of generally accepted threshold for the item-level testing using mean values in the literature, we considered the lowest score option plus 20% of the score range and the highest score option minus 20% of the score range as a practical heuristic to identify outlier items. This resulted in an exclusion criterion for the item score mean of < 1.6 or > 3.4. Any items that met any of these exclusion criteria were removed from the scale.

#### Confirmatory factor analysis

Given that the hypothesized factor structure was specified a priori in part one (i.e., the conceptual model), a confirmatory factor analysis (CFA) was used to establish the construct validity using IBM SPSS AMOS version 24. Model fit was assessed using goodness-of-fit indices, including minimum discrepancy divided by degrees of freedom (CMIN/df), comparative fit index (CFI), root mean square error of approximation (RMSEA), Tucker-Lewis index (TLI), parsimonious normed fit index (PFNI), and parsimonious comparative fit index (PCFI). The criteria for a good fit were: CMIN/df < 3; RMSEA < 0.08; TLI > 0.90; CFI > 0.90; PNFI > 0.50; PCFI > 0.50 [43].

A total of 451 family caregivers participated in the scale validation. Socio-demographic information of participants were described in Table 1.

**Table 1:** Socio-demographic information of participants (*N* = 451)

Variable	Category	*n*	%
Gender	Male	151	33.50
	Female	300	66.50
Age	18–30	277	61.40
	31–40	66	14.60
	41–50	37	8.20
	51–60	48	10.60
	61–70	19	4.20
	71–80	2	0.40
	81 or above	2	0.40
Educational level	No formal education	1	0.20
	Primary school	10	2.20
	Secondary school	63	14.00
	Associate degree	33	7.30
	Bachelor’s degree	246	54.50
	Master/PhD	98	21.70
Marital status	Never married	316	70.10
	Married	127	28.20
	Divorced/separated	6	1.30
	Widowed	2	0.40
Employment status	Full time job	164	36.40
	Part time job	40	8.90
	Unemployed	5	1.10
	Full-time caregiver/housewife	22	4.90
	Retired	20	4.40
	Studying	200	44.30
Family household’s income (in Hong Kong Dollar)	10,000 or below	53	11.80
	10,000–19,999	76	16.90
	20,000–29,999	95	21.10
	30,000–39,999	80	17.70
	40,000–49,999	47	10.40
	50,000–59,999	29	6.40
	60,000 or above	71	15.70

#### Reliability

The internal consistency was evaluated based on Cronbach’s alpha coefficients, which refer to the correlations at an item-level. A Cronbach’s alpha coefficient of ≥ 0.70 would considered an indication of acceptable internal consistency [44]. I Kennedy [45] recommended that a minimum of 100 participants should be sampled to provide a robust assessment of test-retest reliability. Using a random number generator, a random sample of 120 participants from the larger sample pool of scale validation (*N* = 451) were invited to complete the questionnaires two weeks later to assess test-retest reliability [37]. A test-retest correlation coefficient of ≥ 0.50 would be considered that the scale is reliable over time [44].

#### Concurrent validity

To evaluate the concurrent validity of the HLS-FC, we randomly invited half of the participants (*N* = 226), using a random number generator, from the larger sample pool used for scale validation to complete the concurrent validity measure. Based on an a priori power analysis using G*Power, a sample of 84 would be sufficient to detect a medium effect size (*r* = 0.3) in correlational analysis with a power of 80% at *p* < 0.05. As such, we don’t want to overburden the caregivers in completing the full-length survey again. The Chinese version of the HLS-EU-Q47 was utilized to assess concurrent validity [24]. The HLS-EU-47 comprises four information-processing domains (finding, understanding, judging, and applying) and three health domains (health care, disease prevention, and health promotion) that measure HL in the general population. The HLS-EU-Q47 was validated in the Chinese population [46]. The HLS-EU-Q47 was administered immediately after the completion of part two of the validation study [37]. Pearson correlation was computed between the scores of HLS-EU-Q47 and HLS-FC. A positive Pearson correlation of ≥ 0.50 was considered indicative of adequate concurrent validity.

## Results

### Conceptual model and items of HL in FCs of older people

A total of 31 FCs aged between 26 and 89 participated in the focus group, of whom 22 were female. The majority of FCs were providing care for their spouse (*n* = 19), followed by parents (*n* = 11) and grandparents (*n* = 1). Nine had graduated from associate degree, seven had completed primary school, 11 had finished secondary school, three had attended university, and one did not receive formal education. In the second focus group, there were also 31 FCs participated, with seven individuals leaving their age blank, while the others ranged from 27 to 89. Among them, 22 were female, 13 were patients’ children, 18 were spouses, and one was a grandson.

**Fig. 2:**
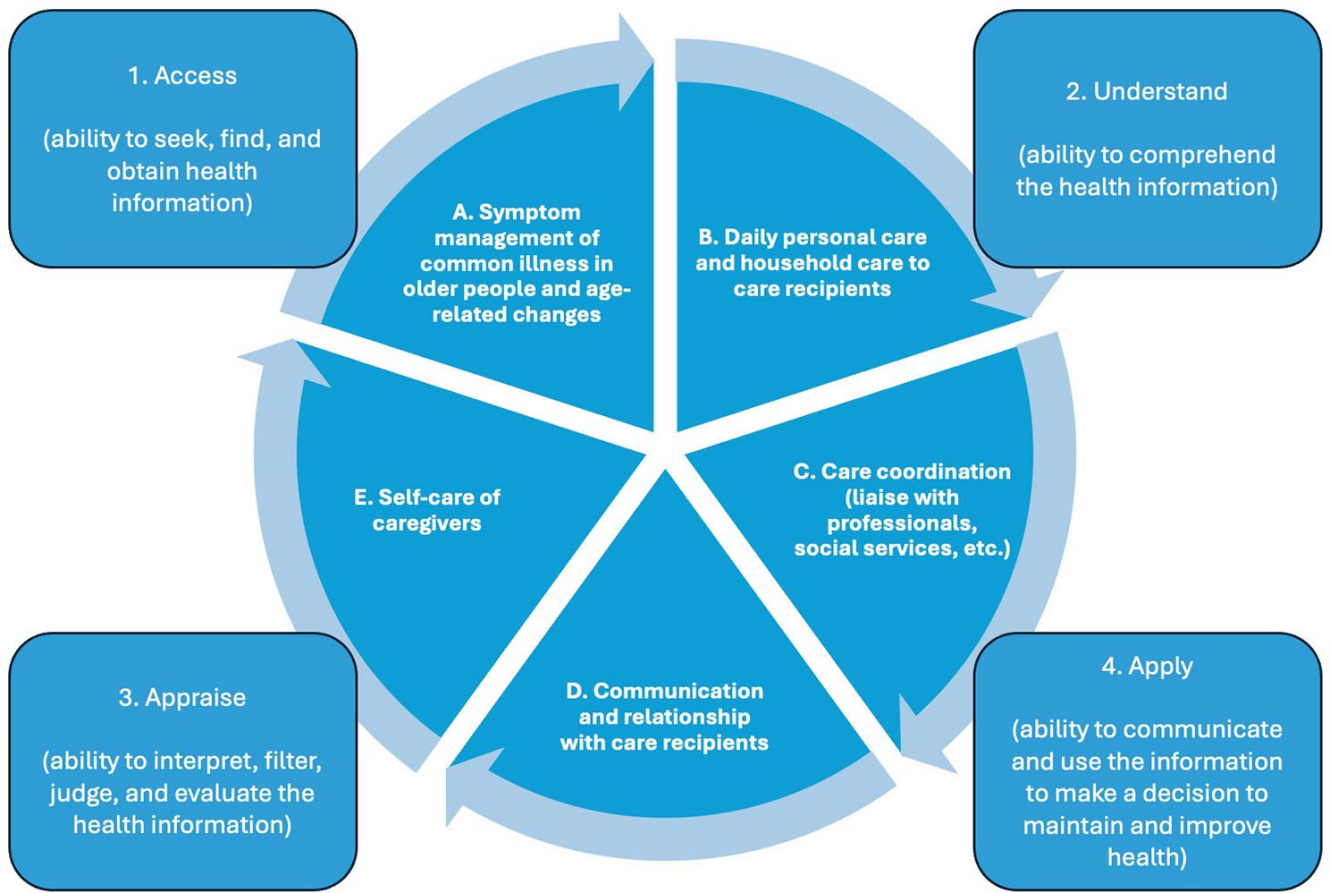
Conceptual model of health literacy among family caregivers of older people with chronic illness

A 20-dimension conceptual model was constructed. The dimensions were formulated by five domains including symptom management, daily care, care coordination, communication, and self-care, encompassing four levels of HL skills (accessing, understanding, appraising, and applying health information) (Fig. 2).

### Item generation

Sixty items were generated from the statement of focus group and conceptual model (developed in part one), with careful consideration of existing instruments on HL and theories of HL.

### Content validity and cognitive interview

Out of the 60 items, eight items, including A2, A6, A 13, A17, B2, B3, B4, B11, were consider redundant by the experts in terms of meaning and described situations, and were therefore deleted. The 52-item scale obtained an overall CVI of 0.90, ranging from 0.85 to 1.00 per item. Cognitive interviews were further conducted to pre-test the instrument. Notably, some of the items were revised due to linguistic errors in Chinese.

### Item reduction analysis

The item reduction process was based on missing rates and mean values. Items that meeting the exclusion criteria were excluded. Out of the 52 remaining items, six items (A3, A9, B5, B14, B16, E4) were excluded. The details were presented in Table 2.

**Table 2:** Item reduction results for 52 items

No.	Item	Missing Rate> 5%	1.6 > M < 3.4	SD
A1	Find information about the diseases affecting the care recipient.	3.80	2.83	0.67
A3	Find information on how to handle emergency medical situations for the care recipient.	6.50	2.65	0.72
A4	Prepare questions before communicating with healthcare professionals.	4.50	2.65	0.69
A5	Understand the health condition of the care recipient as documented in health/medical records or documents (e.g., medication records).	2.90	2.70	0.73
A7	Understand the potential side effects of medications and treatments received by the care recipient.	3.10	2.64	0.71
A8	Understand the explanations given by doctors and nurses about the care recipient’s condition.	2.70	2.77	0.68
A9	Decide when you should seek another doctor’s opinion for the care recipient.	7.40	2.42	0.77
A10	Decide when you should take the care recipient to see a doctor for examination.	3.40	2.65	0.75
A11	Assess the accuracy of information obtained from various media sources (e.g., TV, internet).	2.50	2.49	0.80
A12	Assess the accuracy of information and advice provided by healthcare professionals.	3.60	2.66	0.74
A14	Make decisions for the care recipient based on information provided by doctors.	3.10	2.66	0.73
A15	Organize different health information before making decisions for the care recipient.	1.10	2.67	0.70
A16	Follow the advice on care provided by doctors and nurses.	1.10	2.89	0.72
B1	Find information on personal care for the care recipient (e.g., bathing).	4.30	2.80	0.69
B5	Understand the importance of Chinese culture in the care of older people.	15.00	2.71	0.77
B6	Understand information about making healthy lifestyle choices.	2.70	3.03	0.65
B7	Understand how to control diet or exercise when the care recipient has certain health conditions (e.g., diabetes, hypertension).	2.00	2.81	0.71
B8	Understand the daily activities that the care recipient enjoys.	2.50	2.89	0.72
B9	Understand information on food labels.	2.50	2.80	0.79
B10	Understand the eligibility of the care recipient for vaccination.	3.80	2.55	0.82
B12	Decide which foods may cause illness or allergies in the care recipient.	3.10	2.76	0.80
B13	Decide which vaccines the care recipient should receive.	5.40	2.44	0.90
B14	Use information obtained from the media to make decisions about vaccination.	6.30	2.43	0.87
B15	Use health-related information to prepare food for the care recipient.	1.80	2.91	0.70
B16	Provide care for the care recipient based on cultural norms.	11.70	2.68	0.72
B17	Use health-related information to make decisions on how to maintain a healthy lifestyle for the care recipient.	3.10	2.85	0.65
C1	Find information on existing support services/subsidies (assistance programs) available for the care recipient.	4.00	2.46	0.72
C2	Find information on medical services available for the care recipient.	3.10	2.58	0.73
C3	Find information on the different types of government and non-governmental organizations that provide services for older people.	4.30	2.48	0.75
C4	Understand the application procedures for government subsidies or support programs designed for caregivers or care recipients.	4.30	2.37	0.82
C5	Understand the types of services or programs available to support older people.	5.40	2.39	0.76
C6	Understand the criteria for eligibility for programs or assistance projects supporting older people.	5.40	2.36	0.77
C7	Decide which support services are suitable for the care recipient.	4.00	2.43	0.79
C8	Find someone to talk to when you need help.	5.20	2.52	0.83
D1	Find information on how to effectively communicate with older people.	2.90	2.63	0.68
D2	Find information on how to handle behavioral and emotional issues of the care recipient (e.g., anxiety, wandering).	4.90	2.47	0.74
D3	Understand the communication challenges due to the aging process in older people.	2.50	2.56	0.73
D4	Understand how to maintain a positive relationship with the care recipient.	2.20	2.67	0.75
D5	Respect and accept the decisions and choices of the care recipient.	0.90	2.67	0.71
D6	Decide when to accompany the care recipient.	1.10	2.69	0.75
D7	Maintain a calm and relaxed state when communicating with the care recipient.	1.60	2.53	0.79
E1	Find information on support resources (e.g., assistance programs) or platforms for caregivers.	5.20	2.53	0.70
E2	Find information on managing caregiver stress.	5.80	2.59	0.75
E3	Understand your own health condition and what you can do to maintain good health.	2.90	2.72	0.68
E4	Understand that caregiving can lead to psychological issues that may require assistance from others.	6.10	2.56	0.70
E5	Understand the sources of stress and why you may feel discouraged as a caregiver.	4.00	2.68	0.70
E6	Assess when you are experiencing an excessive burden.	3.80	2.62	0.75
E7	Decide when you should seek assistance in sharing caregiving responsibilities.	3.40	2.60	0.74
E8	Decide when you should reach out to someone (e.g., family member) to share your feelings and thoughts.	2.20	2.69	0.75
E9	Decide when you should take some time to relax.	1.80	2.68	0.74
E10	Make decisions to achieve work-life balance.	1.80	2.47	0.79
E11	Make decisions to avoid emotional distress.	2.90	2.50	0.74

Note. A = symptom management, B = daily care, C = care coordination, D = communication, E = self-care.

### Confirmatory factor analysis

The remaining 46 items were validated in a CFA. The results supported a five-factor structure (symptoms management, daily care, care coordination, communication, and self-care), with satisfactory model fit: CMIN/df = 1.75 (*p* < 0.05), RMSEA = 0.04, TLI = 0.94, CFI = 0.95, PNFI = 0.75, PCFI = 0.80. All factor loadings ranged from 0.57 to 0.84 (Table 3). For the CFA, any item with a factor loading less than 0.50 was excluded. Therefore, we removed four items (A1, A16, B1 and C8) out of 46 items on this basis. The finalized 42 items in the HLS-FC is supplemented (Additional file 1).

**Table 3:** Domains and item loadings in the confirmatory factor analysis

Domain	Item	Loading
Symptom management (9 items)	A4	0.59
	A5	0.64
	A7	0.64
	A8	0.62
	A10	0.73
	A11	0.60
	A12	0.60
	A14	0.66
	A15	0.69
Daily care (9 items)	B6	0.65
	B7	0.65
	B8	0.62
	B9	0.57
	B10	0.60
	B12	0.64
	B13	0.60
	B15	0.64
	B17	0.70
Care coordination (7 items)	C1	0.71
	C2	0.74
	C3	0.79
	C4	0.81
	C5	0.83
	C6	0.84
	C7	0.75
Communication (7 items)	D1	0.75
	D2	0.72
	D3	0.74
	D4	0.76
	D5	0.60
	D6	0.62
	D7	0.65
Self-care (10 items)	E1	0.67
	E2	0.70
	E3	0.62
	E5	0.66
	E6	0.66
	E7	0.67
	E8	0.71
	E9	0.77
	E10	0.68
	E11	0.69

The finalized HLS-FC comprises 42 items designed to measure HL among FCs of older people with chronic illnesses. There are five subscales in the HLS-FC. The symptoms management subscale (9 items) assesses the FC’s skills to assess and comprehend the knowledge required to manage the symptoms of the care recipient. The daily care subscale (9 items) evaluates the FC’s capability to provide day-to-day care to the care recipient. The care coordination subscale (7 items) measures the FC’s capacity to coordinate the care across various healthcare providers and settings for the care recipient. The communication subscale (7 items) evaluates the FC’s skills in effectively communicating with the care recipient. Finally, the self-care subscale (10 items) assesses the FC’s ability to maintain their own health and well-being while caring for the care recipient. Each item is rated on a 4-point Likert scale, ranging from 1 (very difficult) to 4 (very easy). Composite scores can be calculated for all items, reflecting an overall score of HL. Additionally, the respective subscale items can be summed to derive domain-specific scores that reflect the abilities in the respective HL skills. The total score ranges from 42 to 168, with a higher score representing a higher level of HL among the FCs of older people.

### Concurrent validity

A total of 200 FCs completed the HLS-EU-Q47. There was a moderate positive correlation between the HLS-EU-Q47 and the HLS-FC, *r* = 0.67, *p* < 0.01.

### Internal consistency

The internal consistency of HLS-FC was evaluated for each of the domains and the total score (Table 4).

**Table 4:** Internal consistency

Domain	Item	Cronbach’s α
Symptom management (9 items)	A4, A5, A7, A8, A10, A11, A12, A14, A15	0.87
Daily care (9 items)	B6, B7, B8, B9, B10, B12, B13, B15, B17	0.84
Care coordination (7 items)	C1, C2, C3, C4, C5, C6, C7	0.91
Communication (7 items)	D1, D2, D3, D4, D5, D6, D7	0.89
Self-care (10 items)	E1, E2, E3, E5, E6, E7, E8, E9, E10, E11	0.91
Total	All 42 items above	0.96

### Test-retest reliability

A total of 119 participants completed the questionnaires two weeks later (T2) to test the test-retest reliability of HLS-FC (Table 5).

**Table 5:** Test-retest reliability of the HLS-FC (*n* = 119)

Domain	T1		T2		*r*
	M	SD	M	SD	
Symptom management (9 items)	23.75	4.58	23.75	4.59	0.69**
Daily care (9 items)	24.81	4.40	24.60	4.50	0.73**
Care coordination (7 items)	16.92	4.26	16.68	4.09	0.70**
Communication (7 items)	18.05	3.92	17.86	3.72	0.67**
Self-care (10 items)	25.80	5.44	25.62	5.82	0.54**
Total scale (42 items)	107.69	18.34	107.49	20.14	0.77**

Note. T1 = time 1, T2 = two weeks after T1

^*^p < 0.05, ^**^p < 0.01.

## Discussion

This study described the process of developing a conceptual model of HL among FCs of older people with chronic illness and validated a newly developed measure, HLS-FC, to assess levels of self-reported HL within this population. Overall, five domains were identified in the conceptualization of HL among FCs of older people, namely, symptom management, daily personal care and household tasks, care coordination, communication and relationship with the care recipient, and self-care of caregivers. Through developing a comprehensive concept map derived from the focus group interviews with subsequent refinements in the expert reviews, this conceptual model offers good conceptual coverage, incorporating a diverse range of perspectives to reflect a more holistic view of caregivers’ HL.

This conceptual model, in conjunction with the Conceptual Model of Cancer Caregiver Health Literacy [18], supports HL as a multidimensional construct within the context of caregiving. Furthermore, the five identified domains are largely consistent with the Conceptual Model of Caregiver Health Literacy, encompassing similar domains such as care coordination, communication and relationship with the care recipient, and self-care of caregivers. On the other hand, some conceptual distinctions were also revealed, such as symptom management in older individuals and age-related changes. Given the heightened risk of developing multimorbidity in older individuals, symptom management in older individuals and age-related changes may be considered an important domain of HL among FCs of older people [20]. Therefore, this comprehensive conceptual model could serve as the groundwork for future studies in developing relevant evidence-based HL interventions.

To the best of our knowledge, this is the first scale developed to measure HL among FCs of older people with chronic illnesses. Identifying levels of HL is crucial as it enables timely HL interventions. For example, low HL among FCs can compromise care delivery and negatively impact the health outcomes of care recipients [9]. FCs with inadequate HL often struggle to comprehend health information and encounter difficulties in effectively communicating with healthcare professionals, leading to a higher prevalence of undetected health issues among care recipients [10, 11]. Ultimately, unclear and insufficient health information presents an ongoing challenge for FCs in caregiving, affecting the physical, psychological, social, and spiritual well-being of both patients and caregivers [47]. Aligned with the Integrated Conceptual Model of Health Literacy [8], we conceptualized HL among FCs of older individuals as a sequential process within each domain, encompassing the stages of accessing, understanding, appraising, and applying health information. Therefore, the HLS-FC not only facilitates the accurate assessment of evidence-based HL interventions but also enables the identification of vulnerable FCs within specific domains, thereby facilitating tailored interventions to address their specific needs. Nurses, for example, could incorporate the HLS-FC into the routine clinical care of care recipients to quickly identify those FCs who are more vulnerable and provide additional support, such as the dissemination of information and service referrals. The domain-specific scores can be used to further tailor the support required.

Overall, the psychometric properties of the HLS-FC measure were satisfactory. A high CVI of 0.90 was achieved, indicating an excellent level of content validity [48]. This content validity was further reinforced by a good fit of the five-factor structure in the CFA as suggested in our conceptualized model (TLI/CFI > 0.90), conducted with a large sample of 451 FCs of older people. This is in contrast to previous studies focusing on this population, which often rely on smaller sample sizes of approximately 100 individuals [49, 50]. Furthermore, concurrent validity was established through a moderate positive moderate correlation (*r* = 0.67) between the HLS-EU-Q47 and HLS-FC, suggesting that the concept of HL is largely consistent between the general population and the caregiving population. Additionally, the internal consistency of the HLS-FC was excellent, with a Cronbach’s alpha coefficient of 0.96. The reliability was further confirmed by a test-retest reliability coefficient of 0.77 over a two-week period, indicating that the HLS-FC is stable over time.

Compared to generic HL measures like the HLS-EU-Q47 [24], the HLS-FC offers a distinct advantage due to its conceptual coverage of HL for FCs of older people with chronic illnesses. Through focus group interviews and expert reviews, we identified HL concepts specialized for this population. For example, within the domain of care coordination, FCs emphasized the significance of understanding information about available government and non-government organizations that offer services supporting caregiving for older individuals. They also stressed the importance of comprehending their eligibility for various supporting schemes to provide optimal care for their care recipients. Notably, caregivers with adequate support experience lower burden levels and can provide more sustainable care for their recipients than would otherwise be possible [51]. Additionally, FCs identified unique aspects related to communication and the caregiver-care recipient relationship. Common age-related diseases, such as dementia, can pose significant challenges in caregiver-care recipient communication [52], where poor communication between FCs and older individuals can result in caregiver stress and negatively impact care recipient outcomes [53, 54], highlighting the needs for relevant HL interventions for this population. For example, previous studies have reported evidence that caregiver-led multi-sensory cognitive stimulation interventions for older people with dementia can empower caregivers, fostering more positive caregiving experiences and reducing negative attitudes toward care recipients [55, 56]. It is plausible that such interventions could also improve HL among FCs of older people. Therefore, the HLS-FC offers a reliable self-reported outcome measure that is easy to administer for evidence-based interventions tailored to this specific population.

### Limitations

There are some limitations in this study. Previous research has demonstrated that HL is a culturally-bound phenomenon [57, 58]. In this study, we only recruited Chinese FCs of older people, which may have affected the generalizability of the HLS-FC. Given the ongoing influence of Confucianism on Chinese FCs, they tend to actively seek social services to optimize the health of the care recipient [59], a trend also reflected in the conceptualization of HL in our study. Likewise, Western counterparts could emphasize more on the financial and/or legal aspects of HL in the context of caregiving [60]. Despite using purposive sampling to ensure the representativeness of our current sample, many participants in the present study were recruited online. It is conceivable that those with internet access may be more engaged in caregiving, have better information access, higher levels of education, and sufficient incomes, enabling them to optimize the utilization of social services. This could further impact the generalizability of our findings. Therefore, future studies are encouraged to validate the HLS-FC in other cultural contexts with a more socio-demographically diverse sample.

Another potential limitation could be the length of the HLS-FC, which comprises 42 items. Although the HLS-FC has fewer items compared to other HL measures—44 items in the HLQ and 47 items in the HLS-EU-Q—its practical usability could still be limited in certain settings, such as clinical environments, where time constraints are a concern. Therefore, future studies are encouraged to conduct psychometric validations aimed at shortening the scale to increase its clinical utility.

In the present study, we did not attempt to establish a cut-off for the newly developed HLS-FC. This decision was made due to the preliminary nature of the scale validation process, which focused on assessing the underlying factor structure, evaluating the psychometric properties, and providing initial evidence of the scale’s reliability and validity. Future research should focus on conducting larger, confirmatory studies to further validate the scale and establish meaningful cut-off points.

### Conclusions

With an aging population and an increasing prevalence of chronic illness among older individuals, assessing HL is becoming increasingly important for FCs as it can significantly impact the health and psychological outcomes of both caregivers and care recipients. This study presents a comprehensive conceptual model of HL among FCs of older individuals with chronic illness that could provide the groundwork for future studies in developing relevant evidence-based HL interventions. We identified five domains of HL: symptom management, daily personal care and household tasks, care coordination, communication and relationship with the care recipient, and self-care of caregivers. Based on this understanding, we developed the HLS-FC to measure HL among this population. The results indicated satisfactory psychometric properties of the HLS-FC, which offers a flexible, self-reported measure that is easy to administer when assessing HL among FCs of older individuals with chronic illness, which can be utilized to identify caregivers with insufficient health literacy and facilitate timely interventions by healthcare professionals.

### Electronic supplementary material

Below is the link to the electronic supplementary material.

Supplementary Material 1

## Data Availability

The datasets used and/or analyzed during the current study are available from the corresponding author on reasonable request.

## Abbreviations

ADL: activities of daily living
CFA: confirmatory factor analysis
CFI: comparative fit index
CMIN/df: minimum discrepancy divided by degrees of freedom
CVI: content validity index
FC: family caregiver
HL: health literacy
HLCS-C: Health Literacy of Caregivers Scale-Cancer
HLS-EU-Q47: European Health Literacy Questionnaire
IADL: instrumental activities of daily living
IRT: Item Response Theory
MDS: multidimensional scaling
PCFI: parsimonious comparative fit index
PFNI: parsimonious normed fit index
RMSEA: root mean square error of approximation
SD: standard deviation
TLI: Tucker-Lewis index
