# Supplementary material for: Development and validation of a health literacy scale for family caregivers of older people with chronic illness
Source: BMC Nurs. 2024 Jul 1;23:447. doi: 10.1186/s12912-024-02057-x (PMC11218080; doi:10.1186/s12912-024-02057-x)
Supplement: Supplementary file 1 — Supplementary Material 1 [file 12912_2024_2057_MOESM1_ESM.docx]

**Health Literacy Scale-Family Caregiver (HLS-FC)**

To assess your proficiency in accessing, understanding, appraising, and applying health information in caring for older people, please rate the following items based on their level of difficulty for you: 1 = very difficult, 2 = difficult, 3 = easy, 4 = very easy.

| Symptom management | Q1. | Prepare questions before communicating with healthcare professionals. |
| --- | --- | --- |
|  | Q2. | Understand the health condition of the care recipient as documented in health/medical records or documents (e.g., medication records). |
|  | Q3. | Understand the potential side effects of medications and treatments received by the care recipient. |
|  | Q4. | Understand the explanations given by doctors and nurses about the care recipient's condition. |
|  | Q5. | Decide when you should take the care recipient to see a doctor for examination. |
|  | Q6. | Assess the accuracy of information obtained from various media sources (e.g., TV, internet). |
|  | Q7. | Assess the accuracy of information and advice provided by healthcare professionals. |
|  | Q8. | Make decisions for the care recipient based on information provided by doctors. |
|  | Q9. | Organize different health information before making decisions for the care recipient. |
| Daily care | Q10. | Understand information about making healthy lifestyle choices. |
|  | Q11. | Understand how to control diet or exercise when the care recipient has certain health conditions (e.g., diabetes, hypertension). |
|  | Q12. | Understand the daily activities that the care recipient enjoys. |
|  | Q13. | Understand information on food labels. |
|  | Q14. | Understand the eligibility of the care recipient for vaccination. |
|  | Q15. | Decide which foods may cause illness or allergies in the care recipient. |
|  | Q16. | Decide which vaccines the care recipient should receive. |
|  | Q17. | Use health-related information to prepare food for the care recipient. |
|  | Q18. | Use health-related information to make decisions on how to maintain a healthy lifestyle for the care recipient. |
| Care coordination | Q19. | Find information on existing support services/subsidies (assistance programs) available for the care recipient. |
|  | Q20. | Find information on medical services available for the care recipient. |
|  | Q21. | Find information on the different types of government and non-governmental organizations that provide services for older people. |
|  | Q22. | Understand the application procedures for government subsidies or support programs designed for caregivers or care recipients. |
|  | Q23. | Understand the types of services or programs available to support older people. |
|  | Q24. | Understand the criteria for eligibility for programs or assistance projects supporting older people. |
|  | Q25. | Decide which support services are suitable for the care recipient. |
| Communication | Q26. | Find information on how to effectively communicate with older people. |
|  | Q27. | Find information on how to handle behavioral and emotional issues of the care recipient (e.g., anxiety, wandering). |
|  | Q28. | Understand the communication challenges due to the aging process in older people. |
|  | Q29. | Understand how to maintain a positive relationship with the care recipient. |
|  | Q30. | Respect and accept the decisions and choices of the care recipient. |
|  | Q31. | Decide when to accompany the care recipient. |
|  | Q32. | Maintain a calm and relaxed state when communicating with the care recipient. |
| Self-care | Q33. | Find information on support resources (e.g., assistance programs) or platforms for caregivers. |
|  | Q34. | Find information on managing caregiver stress. |
|  | Q35. | Understand your own health condition and what you can do to maintain good health. |
|  | Q36. | Understand the sources of stress and why you may feel discouraged as a caregiver. |
|  | Q37. | Assess when you are experiencing an excessive burden. |
|  | Q38. | Decide when you should seek assistance in sharing caregiving responsibilities. |
|  | Q39. | Decide when you should reach out to someone (e.g., family member) to share your feelings and thoughts. |
|  | Q40. | Decide when you should take some time to relax. |
|  | Q41. | Make decisions to achieve work-life balance. |
|  | Q42. | Make decisions to avoid emotional distress. |
